# Bond-selective transient phase imaging via sensing of the infrared photothermal effect

**DOI:** 10.1038/s41377-019-0224-0

**Published:** 2019-12-11

**Authors:** Delong Zhang, Lu Lan, Yeran Bai, Hassaan Majeed, Mikhail E. Kandel, Gabriel Popescu, Ji-Xin Cheng

**Affiliations:** 1 Department of Biomedical Engineering, Boston University, Boston, MA 02215 USA; 2 Department of Physics, Zhejiang University, Hangzhou, 310028 China; 3 National Laboratory on High Power Laser and Physics, Shanghai, 201800 China; 4 Key Laboratory of High Power Laser and Physics, Shanghai Institute of Optics and Fine Mechanics, Chinese Academy of Sciences, Shanghai, 201800 China; 5 Department of Bioengineering, University of Illinois at Urbana-Champaign, Champaign, IL 61801 USA; 6 Department of Electrical and Computer Engineering, University of Illinois at Urbana-Champaign, Champaign, IL 61801 USA; 7 Department of Electrical & Computer Engineering, Boston University, Boston, MA 02215 USA; 8 Photonics Center, Boston University, Boston, MA 02215 USA

**Keywords:** Phase-contrast microscopy, Infrared spectroscopy

## Abstract

Phase-contrast microscopy converts the phase shift of light passing through a transparent specimen, e.g., a biological cell, into brightness variations in an image. This ability to observe structures without destructive fixation or staining has been widely utilized for applications in materials and life sciences. Despite these advantages, phase-contrast microscopy lacks the ability to reveal molecular information. To address this gap, we developed a bond-selective transient phase (BSTP) imaging technique that excites molecular vibrations by infrared light, resulting in a transient change in phase shift that can be detected by a diffraction phase microscope. By developing a time-gated pump–probe camera system, we demonstrate BSTP imaging of live cells at a 50 Hz frame rate with high spectral fidelity, sub-microsecond temporal resolution, and sub-micron spatial resolution. Our approach paves a new way for spectroscopic imaging investigation in biology and materials science.

## Imaging: probing chemical bonds

A phase imaging technique that provides spectroscopic chemical information could bring new opportunities for studying biology and materials science. Delong Zhang, Lu Lan, and coworkers from Boston, Illinois and Shanghai have developed a scheme called bond-selective transient phase (BSTP) imaging. The approach uses nanosecond pulses of mid-infrared pump laser light to excite molecular vibrations in the sample. The mid-infrared light absorption causes a small temperature change in the sample which results in a change of refractive index and thus a transient phase shift, which is read out by a burst of visible probe pulses and a CMOS camera. Tests with samples including a thin oil film, polyurethane beads, living cells, and the interface between two liquids (olive oil and dimethyl sulfoxide) indicate that BSTP imaging can operate with sub-microsecond temporal resolution and sub-micrometer spatial resolution.

## Introduction

Since Antony van Leuwenhoek’s single lens microscope in the 18th century^1^, optical bright field microscopy has relied on absorption as the main contrast mechanism in an intensity image. Thus, samples with low absorption or scattering, such as biological cells, generate weak intensity modulation and low-contrast images. Nevertheless, transparent samples change the probing light significantly in terms of optical phase delay. By introducing an additional quadrature phase shift between the incident and scattered light, Frits Zernike converted the sample’s phase shift into brightness variation, which allowed the investigation of transparent, unlabeled specimens^2^. Later, *holography* was proposed as an approach to convert phase information into intensity via interference between photons passing through a sample and a reference field^3,4^. Holography has since advanced significantly as digital cameras and powerful computer processors have become readily available^5^. The concept of phase has expanded in the field of imaging, providing the capability of capturing phase images quantitatively. Modern phase imaging can be realized using either holographic^6–13^ or non-holographic approaches^14–17^ and has found broad applications in cellular dynamics and disease diagnosis^18–22^. Recent developments in interferometric microscopy have demonstrated a sensitivity of optical path length down to the sub-nanometer scale^23^, promising a broader range of applications in imaging.

However, the phase of photons passing through a sample is largely insensitive to the chemical composition. Lacking such specificity makes it difficult to apply phase imaging to understand molecular interactions in a complex system. Towards this goal, the integration of phase imaging with fluorescent labeling has been reported^24,25^. However, fluorescent labels have fundamental limitations, including photo-bleaching, perturbation of biological structures, and incapability of labeling small molecules. In contrast, intrinsic molecular bond vibrations can be utilized as a label-free contrast for chemical imaging via either infrared (IR) absorption or Raman scattering spectroscopy. Compared to Raman scattering, IR absorption is a much stronger effect, observed as attenuation of the light by the sample. An IR spectroscopy database of common chemicals was published^26^ as early as 1905. However, because of the long wavelength compared to that of the visible spectrum, direct IR imaging has poor spatial resolution. Moreover, it is difficult to extract the intrinsic absorption property from other attenuation effects, such as scattering and reflection. These disadvantages have limited the potential of IR imaging.

We note that the energy from IR absorption causes a temperature increase in the specimen, which changes its refractive index and thus optical path length via the thermo-optic effect. Such a change can be measured and quantified by phase imaging to provide the intrinsic molecular spectroscopy of the specimen. Based on this concept, we present a bond-selective transient phase (BSTP) microscope that introduces chemical information to phase imaging through pulsed IR light perturbation. The temperature increase caused by IR absorption is transient, dissipating within a few microseconds. However, the current phase imaging apparatus, with an imaging speed of up to a few thousand frames per second^23,27^, is still insufficient to record such transient changes in the phase shift. To address this challenge, we developed a time-gated pump–probe camera system capable of capturing transient phase shift variations. Specifically, by utilizing a sub-microsecond burst of laser pulses to *probe* the transient change in phase shift caused by a nanosecond pulsed mid-IR *pump* laser, we achieved widefield BSTP imaging with sub-microsecond temporal resolution.

## Results

### Principle of BSTP imaging

**Fig. 1:**
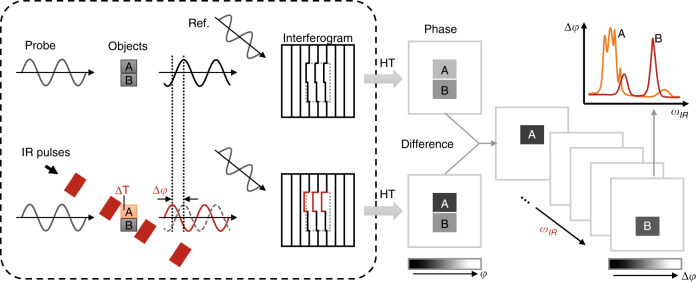
Principle of BSTP imaging. The probe light passes through the sample and interferes with the reference beam at the camera. A phase image of the sample is then retrieved based on the interferogram. Next, IR pulses at the sample induce vibrational absorption (point A) and thus a local temperature increase *ΔT* that changes the local optical phase shift compared to that of point B, which has no absorption peak at this wavelength. The transient change in phase shift *Δφ* is then obtained by subtraction between the adjacent hot and cold phase frames. By tuning the IR frequency *ω*_IR_, the spectroscopy at each pixel can be acquired.

The principle of BSTP imaging is illustrated in Fig. 1. First, using a common-path, off-axis diffraction phase microscope^10^, we generate a quantitative phase image of an unperturbed sample, which is referred to as a “cold” frame. Next, mid-IR pulses illuminate the sample, generating absorption and local changes in temperature, which, in turn, induce a transient change in the refractive index and a modified quantitative phase image. We term this image as a “hot” frame. The phase difference between the hot and cold frames is linearly proportional to the IR absorption at the sample, as shown below.

The optical phase shift *φ* can be described with regard to the refractive index *n* and the thickness *l* of an object in air^28^, as $\varphi = \frac{{2\pi }}{\lambda }(n - 1)l$. Mid-IR pulses with frequency *ω*, energy *E*, and illumination area *A* at the sample are absorbed according to the vibrational absorption coefficient *µ(ω)*, causing a local temperature increase *ΔT*. For simplicity, we assume a single pulse absorption via a steady-state adiabatic process, following the first-order Taylor expansion of the Lambert–Beer law^29^, namely, ${\rm{e}}^{ - \mu (\omega )l} \approx 1 - \mu (\omega )l$. Thus, $\Delta T$ can be expressed as

1 $$\Delta T = \frac{Q}{{C_pm}} = \frac{{(1 - {\rm{e}}^{ - \mu (\omega )l})E}}{{C_p\rho Al}} \approx \frac{{\mu (\omega )E}}{{C_p\rho A}}$$

where *Q* is the amount of heat, *m* is the mass, *C*_*p*_ is the specific heat, $\rho$ is the mass density, and *E* is the mid-IR pulse energy. This local temperature rise results in a change in the refractive index^30^,

2 $$\Delta n = \frac{{{\rm{d}}n}}{{{\rm{d}}T}}\Delta T = \alpha \Delta T$$

and a change in thickness^31^,

3 $$\Delta l = (\frac{1}{l}\frac{{{\rm{d}}l}}{{{\rm{d}}T}})l\Delta T = \beta l\Delta T$$

In Eqs. 2, 3, *α* is the thermo-optic coefficient and *β* is the linear thermal expansion coefficient. The measured change in phase shift, *Δφ*, can be obtained by finding the difference between the hot and cold frames. For small *Δn* and *Δl*, we obtain

4 $$\Delta \varphi = \varphi _{{\rm{hot}}} - \varphi _{{\rm{cold}}} = \frac{{2\pi }}{\lambda }[(n + \Delta n)(l + \Delta l) - nl] = \frac{{2\pi }}{\lambda }(\Delta nl + n\Delta l)$$

Substituting Eqs. 1–3 into Eq. 4, we obtain

5 $$\Delta \varphi (\omega ) = \gamma l\frac{E}{{A\lambda }}\mu (\omega )$$

where $\gamma = \frac{{2\pi (\alpha + n\beta )}}{{C_p\rho }}$. The three factors in Eq. 5 have distinct meanings: *γl* is the physical property of the sample, $\frac{E}{{A\lambda }}$ is the pump pulse property, and *µ(ω)* is the IR spectroscopic absorption. It is clear that *Δφ* is quantitatively related to the chemical content of the sample. Spectroscopic imaging could thus be obtained by tuning the wavelength of a narrow bandwidth IR laser source, providing molecular information to a quantitative phase image.

In BSTP imaging, the absorption-induced heat dissipates immediately when an IR pulse arrives at the sample. Depending on the sample properties, the thermal decay constant ranges from a few microseconds to hundreds of microseconds. Therefore, sub-microsecond temporal resolution is required to detect the transient *Δφ*. However, commercially available cameras are not capable of recording a million frames per second with a sufficient number of pixels for an acceptable interferogram. To address this difficulty, we developed a pump–probe camera system by synchronizing the pulsed IR excitation, pulsed visible probe, and frame capture. In this time-gated scheme, photons reaching the camera are from the probe pulses at a controlled delay from the IR pulses. The thermal decay can be recorded by scanning this delay, providing information about the thermodynamics of the specimen at a temporal resolution determined by the probe pulse width.

### BSTP microscope

**Fig. 2:**
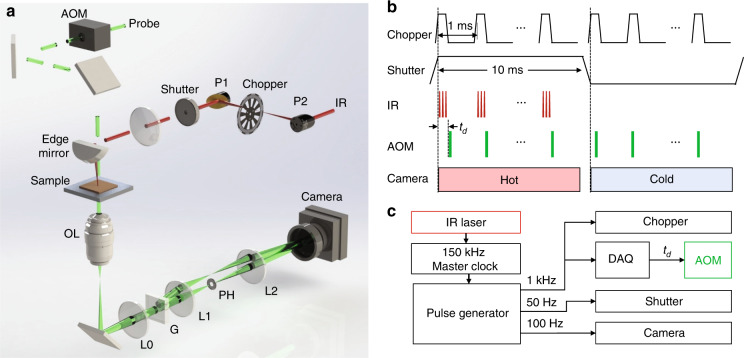
Scheme of the BSTP microscope. **a** Illustration of the BSTP microscope. A common-path diffraction phase microscope was built on an inverted microscope, and an IR beam was introduced for BSTP imaging. AOM: acousto-optical modulator. P1, P2: gold parabolic mirrors. OL: objective lens. L0, L1, L2: lenses. G: grating. PH: pinhole. **b** Timing diagram. *t*_d_: delay of the probe pulse relative to the start of the IR pulses in each burst. **c** Clock hierarchy.

Figure 2a shows our BSTP imaging system. A 520-nm probe pulse train is frequency doubled from an 80-MHz femtosecond pulsed laser at 1040 nm and is chopped by an acousto-optic modulator (AOM) down to bursts of 70 ns duration, which defines the temporal resolution. An important feature of using femtosecond pulses for diffraction phase microscopy is the shorter coherence length, which suppresses the speckle noise when using monochromatic light. A transmission grating was positioned at the conjugate plane of the sample to split the probe beam into multiple orders. Of these orders, we block all but two: the first order, which is filtered by a pinhole at the Fourier plane, and the second order, which passes unaltered^28^. Interference of the two waves, with the first-order wave acting as a reference, generates a stable interferogram at the camera sensor plane. With the IR laser on and off, hot and cold frames are generated and compared to create a BSTP image. A chopper modulates the 150 kHz IR pulse train into 1.0 kHz bursts, minimally a single pulse per burst. Because the interval between IR bursts is set to 1.0 ms and our camera integrates at 10 ms exposure time, we send 9 pump–probe pairs for each camera exposure (Fig. 2b). A shutter closes the IR beam when generating cold frames. The 150-kHz IR laser is used as the master clock, and a pulse generator is used to trigger the chopper, AOM, shutter, and camera (Fig. 2c).

### Characterization of the BSTP imaging signal with a thin oil film

**Fig. 3:**
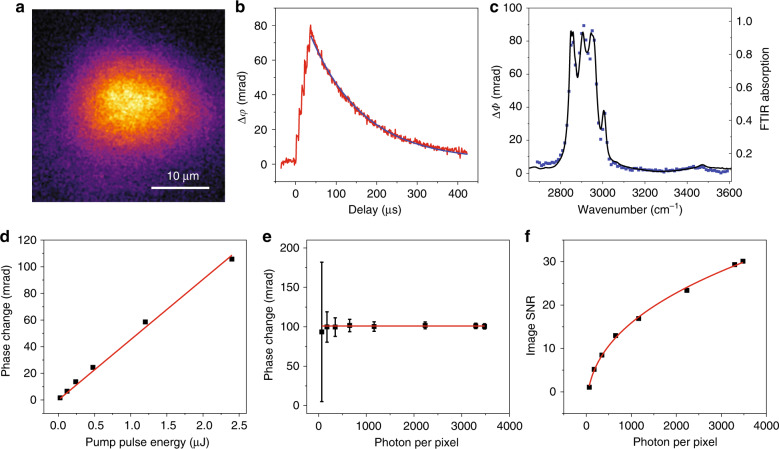
Characterization of the BSTP signal. **a** BSTP image of an oil film sample. There were six IR pulses with 6.67 µs intervals for each image. The IR wavelength was 2950 cm^−1^. **b** Temporal profile of the BSTP signal. **c** Spectral fidelity of the BSTP signal (squares) compared to the standard FTIR spectrum (line). **d** Pump power dependence. BSTP signals at different IR powers (squares) and the linear fitting curve (line). **e** Probe power dependence. BSTP signals with different probe powers (squares) and linear fits (line). **f** Signal-to-noise ratio of BSTP signal with different probe power (squares) and the fitted curve (line).

We measured the temporal profile of the signal from an oil film by scanning the probe pulse delay. The probe width was set at 900 ns, which offers a temporal resolution that, in traditional detection schemes, would only be achievable by a camera acquisition rate of 1.1 million frames per second, significantly above what is commercially available. By tuning the IR pulses to 2950 cm^−1^ for the CH stretching vibration, BSTP images were recorded at each time delay that was tuned electronically by the pulse generator. The temperature increase in oil is estimated to be approximately 1.5 K (detailed in Supplementary Materials). The transient phase images were obtained by subtracting hot phase frames from cold phase frames to create a positive contrast for better visualization. A representative BSTP image is shown in Fig. 3a; the temporal profile is shown in Fig. 3b, i.e., the change in phase shift vs. time delay. In the experiments, 6 IR pulses passed through the chopper within the 1 ms burst cycle. Because of the superior temporal resolution provided by the 900 ns probe pulse, heating by individual IR pulses was clearly resolved for the six pulses with a 6.6 µs interval. Each stepwise increase in the signal corresponded to a nanosecond IR heating pulse. The maximum signal was observed at the end of the 6th IR pulse, followed by a thermal decay with an exponential decay constant τ = 130.8 µs. Notably, this is the slowest decay in our experiments as smaller objects would have much shorter decay constants. To allow sufficient cooling time, a 1.0 ms interval was set between the IR pulse bursts. In the following imaging experiments of beads and cells, the probe pulse was tuned right after the first IR pulse (i.e., 10 ns), allowed for the best temporal resolution and negligible heat dissipation.

To verify that the measured change in phase shift *Δφ* is proportional to the absorption coefficient *µ(ω)*, we scanned the IR wavelength to acquire spectroscopic BSTP images and compared the profile to that from Fourier transform IR (FTIR) spectroscopy (Fig. 3c). The *Δφ* values from each image were plotted along the spectrum of the same sample measured on a standard FTIR microscope, showing good agreement. Furthermore, by fitting the 2910 cm^−1^ peak, a full-width-at-half-maximum of 8.9 cm^−1^ was obtained, which is consistent with the spectral width of 6–9 cm^−1^ for the IR laser. These data demonstrate that BSTP imaging is capable of generating high-fidelity spectroscopic images.

BSTP imaging involves an IR (pump) excitation pulse and a visible probe pulse and can be described as a pump–probe process. It is found that the signal is proportional to the pump power (Fig. 3d), proving a linear absorption by the sample. The BSTP signal level is, however, independent of the probe power (Fig. 3e). This result is different from intensity-based pump–probe measurements. Nevertheless, higher probe power increases the signal-to-noise ratio (SNR) by reducing the noise level in the phase image. Further noise analysis in BSTP revealed the relation between SNR and the number of photons *N* to be ${\rm{SNR}} \propto N^{0.39}$ (Fig. 3f). Ideally, for the shot-noise-limited signal, the power term would be 0.5, according to the Poisson distribution. This faster saturation of the SNR is likely due to mechanical noise in our common-path interferometer. The SNR can be further improved by capturing more photons using stronger probe pulses and a faster camera. Nevertheless, our proof of principle BSTP imaging system is already sensitive to the mrad scale of the IR-absorption-induced change in phase shift.

### Imaging resolution

To characterize the imaging performance of the BSTP microscope, we first studied the spatial resolution of the phase microscope using a standard phase target (Fig. S1). The system was able to consistently resolve 0.78 µm features from phase materials as thin as 50 nm, consistent with the diffraction limit of 0.79 µm for the diffraction phase microscope used here (detailed in Supplementary Materials).

### BSTP imaging of polyurethane beads

**Fig. 4:**
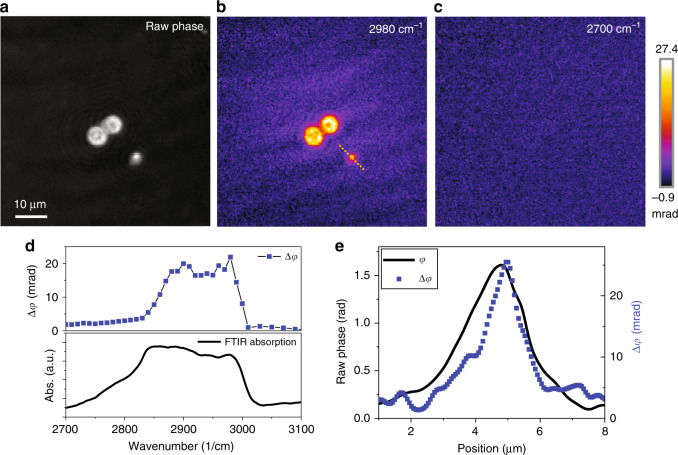
BSTP imaging of polyurethane beads. **a** Quantitative phase image of PU beads. **b**,**c** BSTP imaging of PU beads at an absorption peak of 2980 cm^−1^ and an off-resonance peak at 2700 cm^−1^, respectively. **d** BSTP spectral profile of PU beads (top) and the measured standard FTIR spectrum (bottom). **e** BSTP (blue squares) and raw phase (black line) profiles of the smaller PU bead across the dashed line marked in **b**.

To demonstrate imaging with chemical selectivity, we performed BSTP imaging on polyurethane (PU) beads, providing spectroscopic information at each pixel to augment the conventional phase image (Fig. 4). The bead sample (U7-D50, HOS-Technik GmbH, Austria) has a size distribution of 50% particles at 7 µm. BSTP imaging showed good contrast when the IR laser was tuned to the absorption peak at 2980 cm^−1^ (Fig. 4b), while minimal contrast was observed at an off-resonance frequency of 2700 cm^−1^ (Fig. 4c). We further scanned the pump laser to generate a phase-based IR spectrum of the sample and compared it to the FTIR absorption profile of the same sample (Fig. 4d). Note that the FTIR microscope had difficulty acquiring spectra from single beads due to the IR diffraction limit; it also suffers from sample diffraction artefacts. Therefore, the FTIR spectrum showed an obvious baseline. In contrast, BSTP imaging provides a baseline-free spectrum because the signal only arises from the actual IR absorption. As a result, small characteristic peaks at 2900 and 2980 cm^−1^ were observed in Δφ but were not obvious in the FTIR measurements. We also compared the spatial resolution using a small object in the image. Figure 4e compares the line profiles of the bead between the raw phase and the BSTP images, showing good consistency between them. It is also worth noting that the BSTP images were taken with a single IR pulse at 100 frames per second, i.e., 50 pairs of hot and cold frames, with a 30-frame average, resulting in a speed of 1.67 images per second.

### BSTP imaging of living cells

**Fig. 5:**
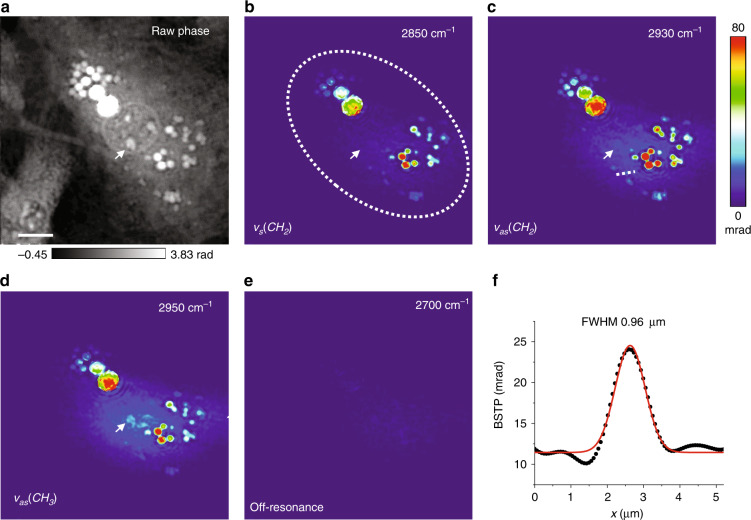
BSTP imaging of live 3T3 cells. **a** Raw phase image of the cells. **b–e** BSTP imaging of 3T3 cells at peaks of 2850, 2930, 2950 cm^−1^, and off-resonance at 2700 cm^−1^, respectively. The IR illumination area is indicated by the dashed circle in (**b**). **f** BSTP intensity profile of a small feature as indicated by the dashed arrow in **c**. Red line: Gaussian fitting result. Scale bar: 10 µm.

To demonstrate the potential of BSTP microscopy for life science, we performed BSTP imaging of live 3T3 cells (Fig. 5). Here, each hot frame was generated by single IR pulse excitation. Nevertheless, the maximum laser energy density that we used at the sample is 11 J/m^2^, which is much lower than the 100 J/m^2^ maximum permissible exposure for cornea according to the ANSI standards^32^. BSTP imaging was performed at various bands, including 2850 cm^−1^ for CH_2_ symmetric stretching, 2930 cm^−1^ for CH_2_ asymmetric stretching, 2950 cm^−1^ for CH_3_ stretching, and 2700 cm^−1^ for off-resonance, as shown in Fig. 5b-e. In addition to the strong lipid droplet signals, the nucleolus showed up at 2950 cm^−1^, mostly contributed by the CH groups in nucleic acids (arrow). At off-resonance, no contrast was observed. Meanwhile, the intensity profile across a small droplet feature (dashed arrow in Fig. 5c) showed a FWHM of 0.96 µm, indicating sub-micron resolution. The result shown in Fig. 5 is intriguing as the cells were living in an aqueous environment. Indeed, the absorption coefficient of water at the OH stretching vibration resonance is larger than 10,000 cm^−1^. Nevertheless, the water absorption coefficient in the CH vibration and the fingerprint window is only 200–600 cm^−1^. Furthermore, the water problem is mitigated by two factors in BSTP imaging. First, for the phase measurement, the IR photons only need to reach the molecules inside a sample. Even at 1% IR power, absorbing molecules would generate a detectable phase shift in the visible probe beam. In this sense, the penetration depth can be over 150 µm at 2700 cm^−1^. Second, water has a high heat capacity, low d*n*/d*T*, and low thermal expansion coefficient, resulting in a small temperature increase and phase shift. As a result, water only generates a *Δφ* value that is 25–80-fold smaller than the *Δφ* from lipids (Fig. 5c, d). Fig S2 shows BSTP imaging of another living 3T3 cell in water with 2930 and 2700 cm^−1^ off-resonance mid-IR excitations. The BSTP images of living cells were taken with a single IR pulse at 100 frames per second, i.e., 50 pairs of hot and cold frames, with a 50-frame average for a high SNR, which leads to an imaging speed of 1 Hz.

### BSTP imaging of the liquid–liquid interface

**Fig. 6:**
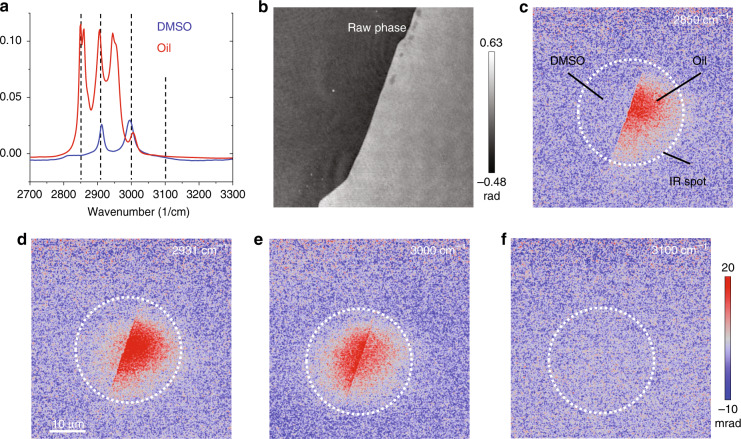
BSTP imaging of the interface between DMSO and oil. **a** Measured FTIR spectra of the DMSO and oil samples. **b** Raw phase image of the interface between DMSO and oil. **c–f** BSTP images of the interface with IR illumination at 2850, 2912, 3000 and 3100 cm^−1^. The dashed circle marks the IR heating spot. Note that the IR beam shifted positions at different wavelengths due to laser pointing; therefore, we adopted a larger field of view in phase imaging to cover the shifting of the IR focus.

To further demonstrate the chemical selectivity, we performed BSTP imaging at the interface between two chemicals, dimethyl sulfoxide (DMSO) and olive oil (Fig. 6). Two small droplets of oil and DMSO were sequentially dropped onto a thin sapphire coverslip of 127 µm thickness to form the interface. The second sapphire coverslip was immediately applied to “sandwich” the oil and DMSO sample. The sandwiched sample was transferred to the microscope, and the interface was identified in the field of view by moving the stage of the microscope. Similar to the PU bead experiments, the opening slit of the chopper was tuned to allow only a single IR pulse pass in each chopper cycle of 1 ms. The probe pulse width was 1.5 µs. Ten pairs of hot and cold images for averaging were applied for imaging at 2850, 2912, and 3100 cm^−1^ and when the IR pulse was blocked. Because the absorption of DMSO was weak at 3000 cm^−1^, averaging of 100 pairs was used.

As shown in Fig. 6, the FTIR peaks of these two compounds overlap. Characteristic peaks are chosen for chemical imaging, namely, 2850, 2912, 3000, and 3100 cm^−1^. At 2850 cm^−1^, only oil has an absorption peak; thus, we detected a strong signal in the recorded image. When the IR laser was tuned to 2912 cm^−1^, signals from both species were present, with the oil peak being more intense. At 3000 cm^−1^, the DMSO peak is stronger than the oil peak, resulting in a higher signal in the image. The *Δφ* image at the off-resonance peak showed a minimal contrast, indicating the spectral fidelity of the system.

Notably, BSTP imaging is not limited to the mid-IR region. The excitation wavelength can be extended to the near-IR window for overtone vibrational spectroscopy. As an example, the overtone absorption of 10-µm poly-methyl-methacrylate (PMMA) beads at a wavelength of 1720 nm provides contrast for BSTP imaging (Fig. S3).

## Discussion

By detecting the optical phase of photons passing through biological samples, phase-contrast microscopy and quantitative phase imaging have become powerful label-free imaging tools to understand biological samples. However, the phase images can only provide refractive-index-based information. This approach is limited in shedding light on the molecular information inside complex biological systems. Here, we have shown that by probing the optical phase change induced by the mid-infrared photothermal (MIP) effect, BSTP imaging offers molecular contrast of phase images without using labels, empowering phase imaging to scrutinize complex biological systems with rich molecular information from vibrational spectroscopy.

We note that two independent studies on the same topic were recently published^33,34^ during the peer review of the present work. Our BSTP work was deposited on arXiv on Nov. 27, 2018 (arXiv:1811.11140), and the aforementioned publications were deposited on Dec. 11, 2018 (arXiv:1812.04576, later published in Scientific Reports), and Mar. 18, 2019 (arXiv:1903.07384, later published in Optics Letters). The fact that these two studies and our BSTP work were independently conducted at essentially the same time adds weight to all studies and shows the paramount importance of adding molecular information to phase imaging. To gain insights for future improvement of the technology, below, we discuss the two key differences between our BSTP approach and the two aforementioned studies.

First, our BSTP microscope provides a nanosecond-scale temporal resolution able to capture a transient phase change induced by IR absorption. In the *Scientific Reports* paper, a continuous-wave IR excitation modulated at 3 kHz was probed by a continuous-wave probe with a camera running at 10 kHz, which provides low temporal resolution of the heating process. In the *Optics Letters* paper, an IR excitation pulse of 5 µs and a visible probe of 130 ns were used, which detected the phase change after 5 µs. In contrast, we deployed a 10 ns IR pulse and a pulsed probe down to 50 ns in BSTP imaging, in which the signal was detected immediately after the 10 ns IR pulse. This nanosecond-scale temporal resolution is critical for molecular phase imaging of living samples in aqueous environments because water dissipates heat faster than many other media, such as oil.

Second, our BSTP microscope is able to probe subtle phase changes induced by IR absorption. For this purpose, we used a probe light source that has a low relative intensity noise (~*−*140 dBc(Hz)-1) and a low coherence length (~30 µm). This is achieved from the second harmonic generation of a 1040-nm, 80-MHz, 100-femtosecond pulsed Ti–sapphire laser. This probe source provides minimal speckle noise in the interferogram image due to its short coherence length and low frame-to-frame noise. As a comparison, a light emitting diode (LED) and a nanosecond laser diode were used in the *Scientific Reports* and *Optics Letters* publications, respectively. Our approach allowed us to demonstrate BSTP imaging of living 3T3 cells in water with an SNR of 100 at 1 Hz at 2930 cm^−1^. Furthermore, by tuning the IR excitation to different spectral peaks, our BSTP imaging showed spectroscopic features of different intracellular species, offering rich molecular information to quantitative phase microscopy. In comparison, the *Scientific Reports* paper showed the imaging of a fixed HeLa cell at a single wavelength of 1530 cm^−1^ with an SNR of 12.6 at 10 Hz. Later, the *Optics Letters* paper demonstrated imaging of 6-µm porous silica beads with an SNR of 5 at 1 Hz. Collectively, these data show the importance of using a quiet probe light source of low coherence length in molecular phase microscopy.

BSTP imaging detects local changes in phase shift induced by mid-IR absorption of chemical bonds and subsequent temperature increase inside a specimen. We note that such a local temperature increase modifies both the physical length and the refractive index of the sample, both contributing to the optical phase shift. While the physical dimension always increases after IR absorption, the refractive index change can be either positive or negative, depending on the material properties. For example, PMMA has a refractive index of ~1.494 and a linear thermal expansion coefficient of *β* = 75 × 10^−6^ K^−1^ but a negative thermo-optic coefficient^35^ of *α* = −130 × 10^−6^ K^−1^. In BSTP imaging, these two terms result in a rather small coefficient (*α* + *nβ*) of −18 × 10^−6^ K^−1^. However, if the physical dimension change and the refractive index change could be recorded separately, the net coefficient |*α*| + |*nβ*| would be 242 × 10^−6^ K^−1^, which promises an approximately 12-fold increase in the signal level. Thus, optical phase tomography techniques^36–38^, which spatially decouple the refractive index and the thickness of the sample, can be used to further enhance the BSTP signals, especially when the thermo-optic coefficient is negative.

We would note that BSTP microscopy as a widefield imaging platform is over three orders of magnitude faster than the recently developed MIP microscopy, which utilizes a visible beam to probe the photothermal effect caused by IR absorption in a point-scanning manner^39–43^. Providing depth-resolution, sub-micron lateral resolution, and micro-molar detection sensitivity, MIP has enabled various applications in biology and materials science, including high-resolution chemical imaging of live cells, live organisms, cellular metabolites, drug molecules in cells and products, and energy materials^39,43–45^. With the much-improved speed, BSTP imaging is expected to enable broader applications in the related fields.

It is intriguing that we observed clear phase contrast from structures in living cells upon mid-infrared light excitation. First, the penetration depth of IR in water is different in BSTP imaging than in IR imaging methods that directly detect changes in IR photons. In BSTP imaging, it indirectly probes the IR-induced changes using visible photons; the IR photons only need to reach the molecules inside a sample. Even at 1% IR power, absorbing molecules generate a detectable signal. In this sense, the penetration depth can be over 150 µm at 2700 cm^−1^, given that the absorption coefficient of water is 200–600 cm^−1^ in the CH vibration and fingerprint region^46^. Second, water provides a weak BSTP signal background in the mid-IR window of interest here. Water has a high heat capacity, low dn/dT, and low thermal expansion coefficient, resulting in a small temperature increase and a phase shift. When taking polyethylene (PE) and water as illustrative examples, their BSTP signals are proportional to their material property *γ* as shown in Eq. 5, and the coefficient γ of water is ~9-fold lower than that of PE (detailed in Supplementary Materials). Note that the difference in the absorption coefficient has not yet been considered.

The performance of the current system is limited by the camera in terms of pixel well depth and frame rate. In this work, we used a scientific-grade complementary metal–oxide–semiconductor (CMOS) camera with a well depth of 30k photoelectrons and a frame rate of 100 frames per second. Both the sensitivity and the imaging speed of our setup can be improved using cameras with higher well depth and faster frame rates. For example, with a CMOS camera of higher well depth, such as 2 million photoelectrons^23^, an eight-fold improvement in sensitivity is expected. Additionally, the imaging speed of our BSTP approach can be further improved by using cameras with higher frame rates. The theoretical imaging speed of our setup is only limited by the thermal relaxation time, which is typically approximately a few tens of microseconds. As a point of reference, in our bead experiments, the decay is as short as 6.9 µs, which is equivalent to approximately 70,000 frames per second.

The spatial resolution of our setup is determined by the quantitative phase imaging setup. A higher NA objective lens will obviously lead to an improved spatial resolution, which can be further pushed beyond the diffraction limit of the probe light. For example, imaging resolution improvement has been reported in quantitative phase imaging employing structured illumination^47^.

Collectively, the reported BSTP microscope enhances the power of phase imaging with spectroscopic insights, which opens a new window for chemical imaging. A comparison of this new modality with FTIR spectroscopy and existing microscopy modalities can be found in Table S1. In this work, we demonstrated a BSTP imaging platform that provides the spectral and temporal dynamics of a specimen with high spectral fidelity and sub-microsecond temporal resolution. We reached a high speed of 50 images per second, limited by the camera. Though we demonstrated the proof of concept using a diffraction phase microscope frame, our pump–probe approach can also be applied to add chemical information to phase imaging using non-interferometric methods. By revealing the chemical composition and thermodynamic information in addition to quantitative phase imaging, BSTP imaging promises wide applications in biology and materials science. Furthermore, by using mid-infrared excitation at the fingerprint region (800–1800 cm^−1^) where the strongest IR spectroscopy modes are located^48^, richer molecular information and stronger BSTP signals will be expected, endowing phase imaging with quantitative chemical information from vibrational spectroscopy to pave a new way for biology and materials research.

## Materials and methods

### BSTP microscope setup

The BSTP microscope (Fig. 2a) was built on an inverted microscope frame (Olympus IX51, Olympus, USA). First, a common-path diffraction phase microscope was built by adding a diffraction phase imaging arm after the tube lens of the microscope. It comprised a transmission grating (110 groves/mm, Edmund Optics, USA), a camera lens L1 of 60 mm focal length (EF‑S Macro 60 mm F/2.8, Canon, USA), a pinhole PH of 15 µm diameter (P15H, Thorlabs, USA), and a second lens L2 of 150 mm focal length (LA1433-A, Thorlabs, USA). The pinhole was placed to filter the 1st order of the diffracted light from the sample at the Fourier plane of L1 to generate the reference light. L2 then combined the unfiltered 2nd order of the diffracted light with the reference light to create interference at the camera. The probe beam was a 520-nm femtosecond pulse train with an 80 MHz repetition rate, which was obtained by frequency doubling the 1040 nm light emitted from a Ti–sapphire femtosecond laser (Chameleon, Coherent, USA). An acousto-optical modulator (AOM, Gooch and Housego) was used to turn on/off the probe beam in synchronization with other parts of the setup.

An IR pulsed laser (Firefly-HW, M-Squared Laser, UK) was used as the pump light. The laser has a pulse width of <10 ns and runs at a repetition rate of 150 kHz. The direct output power from the IR laser used was approximately 61 mW in experiments. A mechanical chopper (MC1F10A, Thorlabs Inc., USA) and a shutter (LS6, Vincent Accessories, USA) were used to open and close the IR beam for imaging. To fit in the small tuneable opening slit of the chopper, a pair of gold parabolic mirrors (MPD149-M01, Thorlabs, USA) was used, and the chopper was placed at the focal plane of the pair of the parabolic mirrors. A calcium fluoride lens with 100 mm focal length was added after the shutter to weakly focus the IR beam at the sample. An edged mirror (PFD10-03-M01, Thorlabs, USA) was used to combine the IR beam at a small oblique angle relative to the probe beam.

### Timing for BSTP imaging

An active monitoring signal from the IR laser provided the primitive master trigger signal for the entire system. The laser runs at a repetition rate of 150 kHz. By using a pulse delay generator (9200, Quantum Composers Inc., USA), we converted the monitoring signal into a 1 kHz master signal to provide a 1 ms period for the thermal relaxation in the specimen in each IR heating cycle, which was sufficient compared to the thermal decay constant of the oil film (130.8 µs) in experiments. This 1 kHz triggering signal was directly sent to trigger both the chopper and a data acquisition card (DAQ, PCIe-6363, National Instrument), which controlled the AOM in the optical path of the probe beam. The duty cycle of the chopper was adjusted to control the number of IR pulses in each chopping cycle. In the BSTP imaging experiments, the number of IR pulses in each chopping cycle was 6 for the oil film, 1 for the PU bead imaging, and 1 for the rest of the experiments. The DAQ was used to set a programmable time delay, *t*_d_, of the probe pulse relative to the start of the IR pulses. For the imaging of PU beads and the DMSO/oil interface, the delay *t*_d_ was set to have the probe pulse start immediately after the IR pulse(s). The pulse width of the probe beam used was 0.9 µs in the experiments of imaging the oil film, 0.4 µs for the cell imaging and 1.5 µs in the rest of experiments. Next, we further down-converted the master 150 kHz trigger to 50 Hz with a 50% duty cycle to switch the shutter on-off and to a 100 Hz TTL signal to match the speed of the camera. By doing so, a pair of adjacent cold and hot phase images were recorded within 20 ms. Considering the dead time of switching on and off the shutter, a 9 ms exposure time was used in all experiments. Figure 2b, c shows the detailed timing diagram for each component in the system.

### BSTP imaging of a thin oil film

A small droplet of olive oil was added on a thin sapphire coverslip of 127 µm thickness. Next, another thin sapphire coverslip of 127 µm thickness was placed on top to sandwich the sample, which was then used for imaging. An objective of ×20 and 0.35 NA was used for the imaging. For the study of the temporal dynamics of the oil film, the IR beam was tuned to 2950 cm^−1^, which is at the C–H stretching vibration in oil. The chopper was adjusted to have a duty cycle of 4%, which corresponds to a total of 6 IR pulses illuminated on the sample. The camera was operated at a speed of 100 Hz, providing 50 pairs of cold and hot phase images per second. No averaging was performed here. By using a customized LabView program on the NI-DAQ card, we scanned the delay *t*_d_ of the probe pulse relative to the starting time of the IR pulses from −40 to 425 µs with a step size of 0.9 µs. For the spectroscopic imaging experiments of the oil film, the same experimental conditions as above were applied except that (1) the delay *t*_d_ of the probe to the IR pulses was fixed at the maximum signal at 40 µs and (2) the wavelength of the IR pulses was scanned from 2700 to 3600 cm^−1^ with a step size of 10 cm^−1^. No averaging of images was conducted. The power of the IR beam at different wavelengths was measured using a power meter and used in the normalization of the measured spectroscopic images by our BSTP microscope.

### BSTP imaging of polyurethane beads

The PU beads (U7-D50, HOS-Technik GmbH, Austria) in powder form were first added to deionized water and then fully dispersed under sonication. A thin film of PU beads on a thin sapphire coverslip of 127 µm thickness was prepared by the drop-casting method. Another small droplet of DMSO solution was then added. The PU beads in the DMSO solution were sandwiched by the placement of a second thin sapphire coverslip of 254 µm thickness. The sandwiched sample was inverted and placed on the microscope for imaging. The opening slit of the chopper was adjusted to allow only one IR pulse to pass in each chopper cycle of 1 ms. The probe pulse used was 1.5 µs. The delay of the probe pulse with respect to the peak of the IR pulse was set to 0. As above, the camera was running at a speed of 100 Hz. An average of 30 pairs of cold-hot phase images was used to obtain the final BSTP images, providing a finalized imaging speed of 1.67 Hz.

### BSTP imaging of living 3T3 cells

Mouse 3T3-L1 preadipocytes (ATCC) were cultured in a basal medium containing high-glucose Dulbecco’s modified eagle medium (ThermoFisher, USA) supplemented with 10% fetal bovine serum (ThermoFisher, USA) and 1% glutamine−penicillin−streptomycin (GPS). To differentiate 3T3 cells into adipocytes, at 2 days’ postconfluence, the basal medium was changed to a differentiation medium composed of a basal medium supplemented with 0.25 μM dexamethasone, 2 μM rosiglitazone, 10 μg/mL insulin, 0.5 mM 3-isobutyl-1-methylxanthine, and 0.2 mM ascorbic acid 2-phosphate (all from Sigma-Aldrich, USA). After another 2 days, the differentiation medium was replaced with a maintenance medium composed of a basal medium supplemented with 10 μg/mL insulin only for 6 days prior to the imaging. The cell culture was done with a customized petri dish that has a thin calcium fluoride window as its bottom. After culturing, the calcium fluoride window that had cells attached and grew on it was detached. It was later sandwiched with another thin calcium fluoride window for the following imaging experiments. The cell culture and imaging procedures followed the protocol (reference number: 17-2256) that were approved by the Boston University’s Institutional Biosafety Committee.

### FTIR spectroscopy measurements

FTIR spectra of oil, DMSO, and PU beads were measured using a commercial FTIR system (Hyperion, Bruker) in reflected mode. Small droplets of oil and DMSO were dropped on two gold mirrors, and a thin film of PU beads from the same suspension above was formed on another gold mirror using the same drop-casting method. Later, all three samples were sandwiched by adding a 1 mm thick calcium fluoride window on the top and then transferred to the FTIR system for imaging. The FTIR spectra of all three were measured from 500 to 4000 cm^−1^ with a resolution of 4 cm^−1^.

## Supplementary information

Supplementary Information

## Data Availability

The authors declare that all of the data supporting the findings of this study are available within the paper and the supplementary information.
