# Supplementary material for: Bond-selective transient phase imaging via sensing of the infrared photothermal effect
Source: Light Sci Appl. 2019 Dec 11;8:116. doi: 10.1038/s41377-019-0224-0 (PMC6904725; doi:10.1038/s41377-019-0224-0)
Supplement: Supplementary file 1 — Supplementary Information [file 41377_2019_224_MOESM1_ESM.docx]

Supplementary materials for

**Bond-Selective Transient Phase Imaging via Sensing of the Infrared Photothermal Effect**

Delong Zhang†^1,2^, Lu Lan†^1^, Yeran Bai^1,3,4^, Hassaan Majeed^5^,

Mikhail E. Kandel^6^, Gabriel Popescu^5^,^6*^, Ji-Xin Cheng^1,7,8^*

1. Department of Biomedical Engineering, Boston University, Boston, MA 02215, USA

2. Department of Physics, Zhejiang University, Hangzhou, 310028, China

3. National Laboratory on High Power Laser and Physics, Shanghai 201800, China

4. Key Laboratory of High Power Laser and Physics, Shanghai Institute of Optics and Fine Mechanics, Chinese Academy of Sciences, Shanghai 201800, China

5. Department of Bioengineering, University of Illinois at Urbana-Champaign, Champaign, IL 61801, USA

6. Department of Electrical and Computer Engineering, University of Illinois at Urbana-Champaign, Champaign, IL 61801, USA

7. Department of Electrical & Computer Engineering, Boston University, Boston, MA 02215, USA

8. Photonics Center, Boston University, Boston, MA 02215, USA

†Authors contributed equally to this work

Correspondence to: [jxcheng@bu.edu](mailto:jxcheng@bu.edu), [gpopescu@illinois.edu](mailto:gpopescu@illinois.edu)

**1. Performance of the quantitative phase microscope**

To characterize the performance of the phase microscope, we imaged a standard phase target (Benchmark Technologies) in 1951 USAF resolution test pattern (**Fig. S1**). Our phase microscope was able to image the phase material as thin as 50 nm without averaging. The system was able to consistently resolve 0.78 µm features (group 9 element 3). The lateral resolution is determined by the quantitative phase microscope, which is given by the Abbe’s formula 1.22*λ*/(*NA_obj_* + *NA_con_*) ≈ 1.22*λ/NA_obj_*, where *NA_obj_* is the numerical aperture of the objective, and condenser numerical aperture *NA_con_* ≈ 0 for typical plane wave illumination. Plug in *λ* = 520 nm, objective *NA* = 0.8, we have a resolution = 0.79 µm, which is consistent with the experimental value.

**Figure S1. Phase imaging of USAF Resolution targets.** The targets were made with transparent materials in thickness of 300 nm (**a**), 150 nm (**b**), and 50 nm (**c**).

**2. Degree of temperature increase at the sample**

We assume no heat dissipation and use the following equation

$\Delta T=\frac{Q}{mC_{p}}=\frac{Q}{\rho AdC_{p}}$,

where *Q* is the absorbed IR energy, *ρ* is oil density, *A* is the area of IR illumination, *d* is sample thickness, *Cp* is the specific heat, m is the mass of oil, and *ΔT* is the change in temperature. The total IR transmission of the system is measured to be 17% after a clean-up filter, an uncoated CaF_2_ beam sampler, and 12 gold surfaces. Therefore, for 0.67 µJ pulse out of the laser head, about 0.11 µJ reaches the sample. The IR spot size is about 110 µm, calculated from 4 mm beam diameter and 100 mm focusing lens (effective focal length 105 mm at 3300 nm). Therefore, the laser energy density is 11.3 J/m^2^. The absorption coefficient of oil at 2950 cm^−1^ is estimated to be 3000/cm, therefore 90% of the IR energy went into oil after 3.3 µm. Plug in *ρ* = 880 kg/m^3^ and *C_p_* = 2300 J/kgK, we have *ΔT* = 1.5 K. Note that this number is under the assumption of no heat conduction, therefore is the upper limit of the temperature rise, and in real samples, such heat usually dissipates in a few microseconds.

**3. BSTP imaging of living 3T3 cell #2
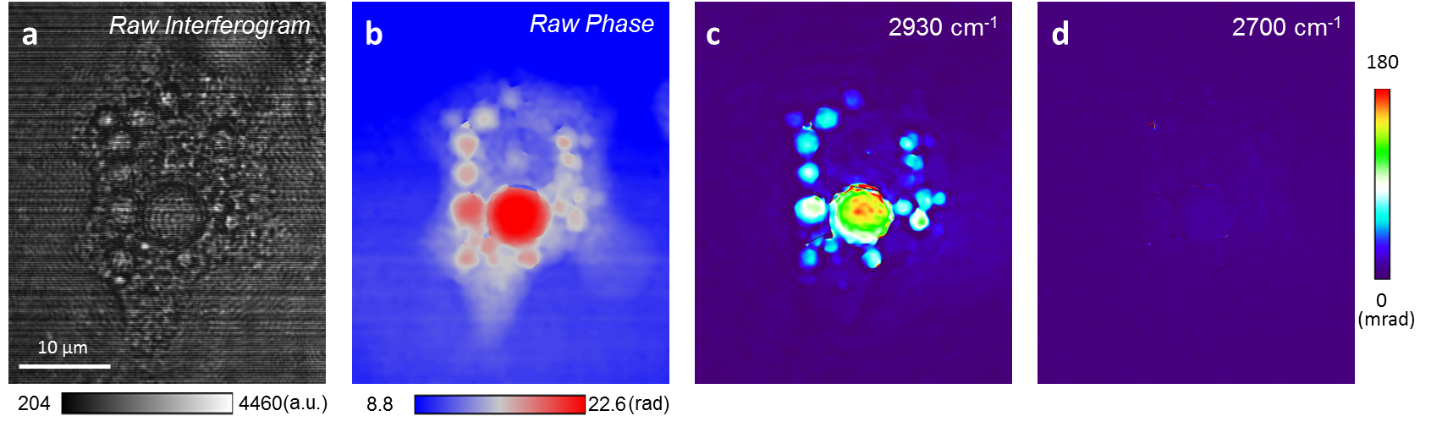
**

**Figure S2. BSTP imaging of living 3T3 cell #2.** **a** Raw Interferogram of a living 3T3 cell #2, **b** Raw phase image of the living 3T3 cell #2. **c, d** BSTP imaging of the living 3T3 cell #2 at peaks of 2930 cm^-1^, and off-resonance at 2700 cm^-1^, respectively.

**4. BSTP imaging in the near-infrared window**

The overtone absorption of 10-µm Poly-methyl-methacrylate (PMMA) beads provides contrast for BSTP imaging (**Fig. S3**). The 1720 nm second overtone absorption generated a phase difference compared to that at the 1520 nm non-resonant wavelength.

**Figure S3. BSTP imaging of 10-µm PMMA beads with near-infrared excitation.** **a**, Raw phase image after 1520 nm excitation pulse. **b**, Raw phase image after 1720 nm excitation. **c**, Near-IR absorption spectrum of PMMA. **d**, BSTP image of PMMA beads at 1720 nm excitation.

**5. Effect of water absorption.**

We compared polyethylene (PE) and water as an example to illustrate the effect of water to BSTP signal. As shown in the manuscript, the bond-selective phase transient signal is proportional to$\gamma=\frac{2\pi(\alpha+n\beta)}{C_{p}\rho}$, in which the thermo-optic coefficient *α* = *dn/dT*, and the linear thermal expansion coefficient $\beta=\frac{1}{l}\frac{dl}{dT}$. Plug in the physical constants listed in the table below ^1^, we have the coefficient *γ* of water ~9 times lower than that of PE. Note that the difference of absorption coefficient has not yet been considered.

|  | *C_p_* (J/kgK) | *ρ* (g/cm^3^) | *β* (10^-6^/K) | *α* (10^-6^/K) | *n* |
| --- | --- | --- | --- | --- | --- |
| Polyethylene | 1300~1500 | 0.975 | 108~200 | −130 | 1.5 |
| Water | 4200 | 1 | 51 | −100 | 1.33 |

**6. Comparison of BSTP imaging with existing microscope modalities.**

Performance characteristics of BSTP and related microscope modalities are shown in the table below. It is seen that each technique has its own strength and limitations, therefore is applicable to different scenarios. BSTP microscope enhances the power of phase imaging with spectroscopy insights, which opens a new window for chemical imaging.

| **Modality** | **Chemical selectivity** | **Spatial resolution** | **Imaging speed** | **Temporal resolution** |
| --- | --- | --- | --- | --- |
| **BSTP microscope** | Fingerprint | Sub-micron | 50 fps | Nanosecond level |
| **FTIR microscope** | Fingerprint | ~10 µm | <0.1 fps | Minute level |
| **Fluorescence microscope** | Depending on labeling | Typ. ~0.3 µm, up to 50 nm | Up to 1000s fps | Nanosecond level |
| **Pump-probe microscope** | Only for electronic absorption | Up to ~0.3 µm | Video rate | Femto- to picosecond |
| **Phase microscope** | No | Up to ~0.3 µm | Video rate | Microsecond level |

**Table S1. Characteristics of BSDP microscope versus existing microscope modalities.**

**References**

1 Burnett, J. H. & Kaplan, S. G. Measurement of the refractive index and thermo-optic coefficient of water near 193 nm. Vol. 3 (SPIE, 2004).
